# The Relationship Between Psychological Quality Education and Mental Health Level of College Students by Educational Psychology

**DOI:** 10.3389/fpsyg.2022.892143

**Published:** 2022-06-14

**Authors:** Ruihong Xu

**Affiliations:** School of Marxism, Hunan University, Changsha, China

**Keywords:** educational psychology, psychological quality education, mental health level, questionnaire, college students

## Abstract

To enable all college students to have positive psychological quality and sound personalities, their potential should be fully realized, and their comprehensive ability should be improved to adapt to society. Empirical research is carried out by means of questionnaires, and the relationship between psychological quality education and the mental health level of college students is studied through correlation analysis and regression analysis. Firstly, the problems existing in college students’ psychological quality education are summarized from the perspective of educational psychology through questionnaires. Secondly, the data of college students’ psychological quality education and mental health level are collected, and the general situation and the relationship between college students’ psychological quality education and mental health level are analyzed and discussed by processing the existing data. The research results show that 51% of college students think that psychological quality education is only needed when there is a psychological problem; 47% of college students believe that the current educational content of the school’s psychological quality education focuses on the prevention and solution of students’ psychological problems; 83% of the students consider that the psychological quality education currently carried out by the college still adopts the more traditional teaching methods such as classroom lectures, psychological counseling and special lectures. In the process of predicting college students’ mental health level, psychological resilience plays a significant role, which can predict 21.1% of variables. Psychological resilience and optimism can jointly predict 26.4% of variables. These contents broaden the research field of college students’ psychological quality education, enrich the related research on it, and provide a reference for the intervention of college students’ psychological health in school situations.

## Introduction

Psychological quality education for college students is an important part of ideological education. Research on college students’ psychological quality and mental health is a problem that the country attaches great importance to. At the symposium on philosophy and social science in 2016, general secretary Jinping Xi pointed out that it was necessary to help all students to improve their moral thinking and spiritual realm, so that they can form correct values, outlook on life and world outlook, develop a scientific thinking mode, and promote comprehensive development of their physical and mental health (Ying et al., 2017; Wang, 2021). In 2017, the Chinese government formulated the document “Guidance on Strengthening Mental Health Services” for mental health work, which is the first document formulated by the government to address this issue (Tian and Tsai, 2020; Liu, 2021). Mental health has been paid attention to by the country. The key step in implementing the policies of the party and the country is to strengthen the cultivation of college students’ mental health. The psychological quality education of college students has been further improved and developed under the new social conditions, and the development of educational psychology has also injected new vitality into mental health education (Burns, 2018; Hoyt et al., 2021).

Foreign scholars have long been committed to the study of the psychological perspective, resulting in a large number of theoretical research results. The concept of “positive qualities” was first proposed by Made and Hillson in 1999. Based on previous research on positive psychology, “Father of Positive Psychology” Seligman published the book “Introduction to Positive Psychology” in 2000. In the book, he pointed out that optimism and happiness were all positive personality traits. Then, in 2002, he proposed that virtue and strength were at the core of positive qualities of the individual, have the role of a buffer, and can help individuals overcome mental illness (Kareen et al., 2018; Chung et al., 2020; Leen et al., 2021). Scholars have proved through a large number of empirical studies that acquired training is conducive to students obtaining positive psychological quality. Relevant scholars indicated that the positive psychological quality of individuals can be cultivated from positive emotions, so that individuals can feel the positive impact of positive emotions, and then develop positive psychological quality in life (Bebber et al., 2018).

However, in the current research, theoretical research is the main research. There is less empirical research, more repetitive research in a certain direction, a lack of innovation in theory and method, and the breadth and depth of the research still need to be expanded. It takes educational informatics as the research perspective and pays attention to exploring the positive psychological quality of individuals. Traditional college students’ mental health education takes negative treatment as the perspective, but it breaks this shackle. The research content has been updated and the perspective has been expanded.

## Research Theory and Method

### Principles of Psychological Quality Education for College Students From the Perspective of Educational Psychology

#### The Principle of Subjectivity

The principle of subjectivity means that the starting point and center of psychological quality education for college students must be students by educational psychology. Students should be the main body. The application of teaching methods and the selection of teaching content in the process of teaching should be based on the premise of respecting the needs of students’ growth, and give full play to the subjective initiative of students. Firstly, the level of mental health is an internal characteristic of students and has a tendency. Under the impetus of self-consciousness, college students selectively receive education and internalize their recognized behaviors into their own internal qualities (Permata et al., 2019; Ricci et al., 2019). The education and teaching process should fully respect the personality of students, so that students can actively participate in the classroom process and practical activities of psychological quality education, and promote the transformation from external education to internal quality. Secondly, psychological quality education needs to combine self-help with other help. The development of college students’ mental health level is essentially a process of self-realization. In the process of participating in educational activities, college students must play a subjective role to achieve the goal of self-realization (West, 2019). If there is a lack of subjective experience during the educational process, educational activities will lose their own meaning and become a coercive act. College students’ independent consciousness and self-awareness are constantly enhanced. To develop their mental health level, they must pay attention to the implementation of the principle of subjectivity in the process of psychological quality education.

#### The Principle of Incentive

Individuals’ behavioral activities are based on needs. Only when individuals have needs for things will they stimulat their behavioral motivation, resulting in behavior (Jia et al., 2019). Marx once pointed out, “Everything that people struggle is related to their interests.” If the psychological quality education of college students can meet the reasonable needs of students, it can stimulate students’ enthusiasm and avoid losing their educational foothold due to big talk. The educational process should focus on incentives, stimulate students’ learning motivation and realize their internal potential by meeting their reasonable needs (Ong et al., 2018; Chen et al., 2019; Han et al., 2020). Incentives mainly include spiritual incentives and material incentives. Educators should choose different incentive methods according to the actual situation and personal characteristics of students, and use incentive methods scientifically.

#### The Principle of Experience

It is an essential and crucial step to participate in practical activities for the development of individual ability. Therefore, in the process of college students’ psychological quality education, educators should adhere to promoting the development of college students’ mental health through practical activities. In practical activities, college students can recognize themselves, express themselves, and realize their value and potential (Gao, 2021; Inguva et al., 2021). Modern psychology theory believes that the cultivation of human psychological activities is inseparable from the objective world. The interaction between subject and object forms cognition, and the bridge of cognition is behavioral activities. Psychological quality education aims to cultivate the sound personality and healthy psychology of college students. Achieving this goal requires not only education, but also attention to students’ self-experience. It is necessary to adhere to the combination of theoretical education and practical cultivation, and attach importance to the implementation of the principle of experience in all aspects of education (Beattie et al., 2020). Through the creation of actual situations and practical exercises, students can gain insights during the process of practice, fully understand the theoretical knowledge they have learned, and consciously carry out self-education.

#### The Principle of Development

Materialists think that everything in the world is in the process of continuous development. The principle of development mainly includes the following two aspects: Firstly, college students are people in the process of development and have great development potential. The development of mental health levels is a dynamic process. Most of the psychological problems of college students are non-obstacles. Therefore, educators should view the psychological problems of college students from a development perspective, analyze the problems they face through multiple angles, and make positive interpretations. Secondly, development and correction are relative (Eicher et al., 2018; Solomon et al., 2021). From the perspective of educational psychology, the focus of college students’ psychological quality education is to develop the potential ability of college students, and put the prevention and treatment of mental diseases in an auxiliary position. It is more inclined to pursue development while preventing and treating mental diseases. Only by placing development in an important position, can the mental health of college students be truly improved (Burggren, 2021).

#### The Principle of Infectiousness

The principle of infectiousness refers to the influence of college students through the environment or object under unconscious circumstances, to achieve the purpose of education. Image infection and emotional infection are important forms of infection. Among them, image infection refers to the use of objective and specific things to have a certain impact on students, such as strengthening the environment within the campus (Krueger et al., 2019). Emotional infection refers to the creation of a situational atmosphere, which has a certain influence on students’ emotions and makes them receive educational content, such as visiting the school history museum, carrying out recreational activities, creating a warm family atmosphere, etc. Psychological research has proved that it is more conducive to receiving and understanding knowledge in a comfortable mood (Prata et al., 2018; Ge, 2020). By creating a comfortable environment and enriching practical activities, the educated can maintain a happy mood in the process of receiving education.

Foreign scholars define positive psychological quality as a relatively stable positive psychological quality gradually formed under the interaction of innate ability and acquired environment and educational influence. In China, the earliest definition of positive psychological quality is that it is a relatively persistent and positive subjective emotion and experience, including satisfaction, happiness, pride, excitement, etc. Meanwhile, a structure of 15 qualities in six dimensions of positive psychological quality of Chinese college students is constructed, which deepens the definition of positive psychological quality in operation. These include: cognitive dimension—creativity, intellectual curiosity, thinking and observation; emotional dimension - sincerity, persistence; interpersonal dimension—love, friendliness; civic dimension—leadership, cooperation; moderation dimension—tolerance, humility, prudence; transcendence dimension—heart touching, humor, faith and hope. According to the current research on the psychological quality education of college students, the following hypotheses are put forward:

H1: Psychological quality education will promote the development of college students’ mental health;
H2: Psychological resilience and optimism play a mediating role between the psychological quality education and the mental health level of college students.

### Research Design

#### The Current Situation of College Students’ Psychological Quality Education From the Perspective of Educational Psychology

Currently, the breadth and depth of research on the mental health level of college students based on educational psychology still need to be expanded. To improve the effectiveness and scientificity of college students’ psychological quality education, the problems existing in the current college students’ psychological quality education are studied through a questionnaire, and the reasons for the problems are analyzed (Jing et al., 2018).

##### Subjects

A combination of random sampling and stratified sampling was adopted. Three-hundred students were randomly selected from each grade of Xi’an Technological University, Xi’an University of Architecture and Technology, and Xi’an Polytechnic University in Shaanxi Province for a questionnaire survey. Questionnaires were distributed to them through the “Questionnaire Star” platform, and a total of 300 copies were collected. After excluding the students who answered the questionnaire incompletely and obviously did not answer seriously, 281 valid questionnaires were finally recovered, with an effective rate of 94%, including 138 for boys and 143 for girls, and the ratio of males and females was basically the same.

##### Design of the Questionnaire

After research on educational psychology and college students’ psychological quality education, according to the research content, a questionnaire is designed centered on the current methods, contents, concepts, effects and other content of college students’ psychological quality education. The questionnaire includes two parts: the information of the respondents and the current situation of college students’ psychological quality education from the perspective of educational psychology, with a total of 21 questions, all of which are objective multiple-choice questions, of which the first part has three questions and the second part has 18 questions. Respondents can choose from four options: uncertain, non-conforming, relatively conforming, and fully conforming according to their actual situation. In the statistical analysis of the questionnaire results, uncertain and non-conforming are regarded as negative attitudes, and relatively conforming and fully conforming are regarded as positive attitudes (Wang et al., 2019).

#### The Relationship Between Psychological Quality Education and Mental Health Level of College Students

In recent years, there have been endless mental health problems for college students. Meanwhile, adolescence is also a frequent period of various mental health problems. The mental health problems of college students have attracted the attention of scholars. The general situation and relationship between the psychological quality education and the mental health level of college students are investigated and analyzed mainly through questionnaires.

##### Subjects

A total of 400 college students are randomly selected as subjects from the freshman, sophomore, junior and senior grades of Xi’an University of Architecture and Technology, and a questionnaire is carried out on them. Finally, 383 valid questionnaires are withdrawn, and the questionnaire efficiency is 95.7%. Through the statistics of the basic situation of the subjects, it is found that there are 196 males and 187 females in the subjects, and the proportion of males and females is basically the same. 31% of the students are from rural or urban areas, and the rest of the subjects are urban registered residences. 63% are the only child, and 11% are single-parent families.

##### Design of the Questionnaire

The Symptom Checklist 90 (SCL-90) compiled by Derogatis was used to investigate the mental health level of college students, and the mental health status of college students was investigated from the perspectives of interpersonal relationship, diet and sleep, consciousness, emotion and so on. SCL-90 includes 10 factors, including diet and sleep, somatization, anxiety, obsessive-compulsive symptoms, psychosis, interpersonal sensitivity, terror, hostility, paranoia, and depression, with a total of 90 items. Subjects chose from the following five options according to their own situation: 0–1 indicates asymptomatic, 1–2 indicates uncommon symptoms, 2–3 indicates mild to moderate symptoms, 3–4 indicates moderately severe symptoms, and >4 indicates severe symptoms.

The Cronbach coefficient of SCL-90 is 0.95, and the correlation coefficient between the total scale and the sub scale is 0.75–0.92, indicating that SCL-90 has good consistency and discrimination effectiveness. The overall confidence level is 95%, and the set sample error is ±1.

Positive psychological capital questionnaire is used to investigate the psychological quality of college students. The questionnaire contains four dimensions of resilience, hope, optimism, and self-efficacy, with a total of 26 questions. In the questionnaire, 0–3 indicates that the psychological quality of college students is in a negative state, 4–5 indicates that the psychological quality of college students is general, and >5 indicates that the psychological quality of college students is in a positive state. The Cronbach coefficient of the positive psychological capital questionnaire is 0.9, and the correlation coefficient between the total scale and the sub scale is 0.76–0.96, indicating that the scale has good consistency and discrimination effectiveness. The overall confidence level is 95%, and the set sample error is ±1.

## Analysis of Survey Results

### Analysis of the Current Situation of College Students’ Psychological Quality Education

In recent years, colleges have paid more attention to psychological quality education. Psychological quality education is set as a compulsory course in many colleges and given certain credits, thus enhancing students’ attention to psychological quality education. Some colleges can integrate the relevant content of educational psychology into the process of college students’ quality education, effectively improving the mental health level of college students. However, through this survey, it is found that there are still the following problems in quality education for college students based on educational psychology.

#### Backward Educational Concepts

**FIGURE 1:**
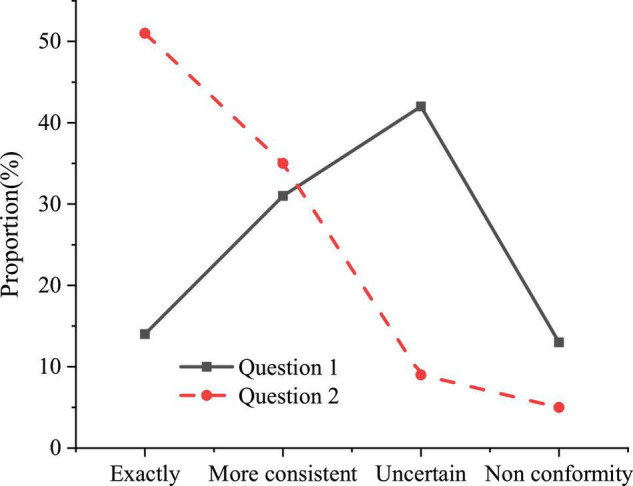
The statistical results of the questionnaire on educational concepts.

The concept of education is the forerunner of educational activities. To establish a positive educational concept in the process of psychological quality education, not only focus on solving students’ psychological problems, but also focus on exploring students’ excellent psychological quality. The statistical results of the questionnaire on educational concepts are shown in Figure 1.

Question 1 refers to “the current educational concept of psychological quality education set up by the colleges is positive” and Question 2 refers to “psychological quality education is only needed when there is a psychological problem.” Figure 1 shows that the educational concept of psychological quality education in colleges is relatively backward. More than half of college students have a negative attitude toward the educational concept of psychological quality education in colleges, and 51% of college students think that psychological quality education is only needed when there is a psychological problem.

#### Outdated Educational Contents

**FIGURE 2:**
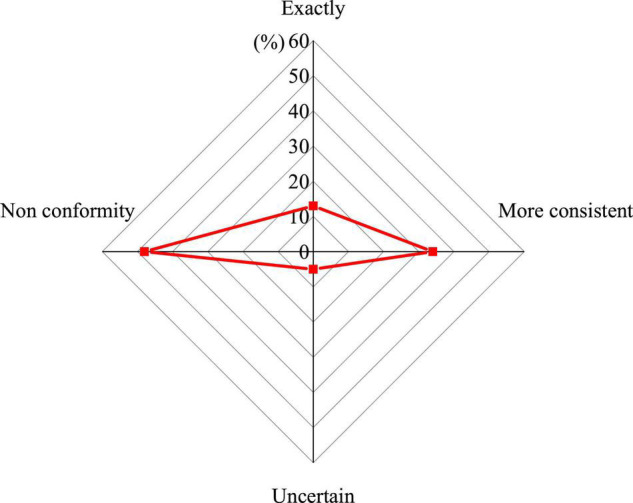
Educational content focuses on the prevention and resolution of students’ psychological problems.

Psychological quality education should include a variety of educational contents such as will cultivation, emotional management and interpersonal relationships. The results of the questionnaire on the content of psychological quality education in schools are shown in Figure 2.

In Figure 2, 47% of college students believe that the educational content of the current colleges’ psychological quality education focuses on the prevention and resolution of students’ psychological problems. During the actual teaching process, most educators will list the psychological problems that students may have, and then interpret and analyze these psychological problems, so that students can solve some psychological problems that may occur later according to their own actual situation. Psychological publicity activities also tend to guide students so that they can pay attention to some of their own psychological problems, but this educational content is too old-fashioned, does not take into account the actual needs of students, and cannot effectively improve mental health level of college students.

#### Lack of Novelty in Teaching Methods

**FIGURE 3:**
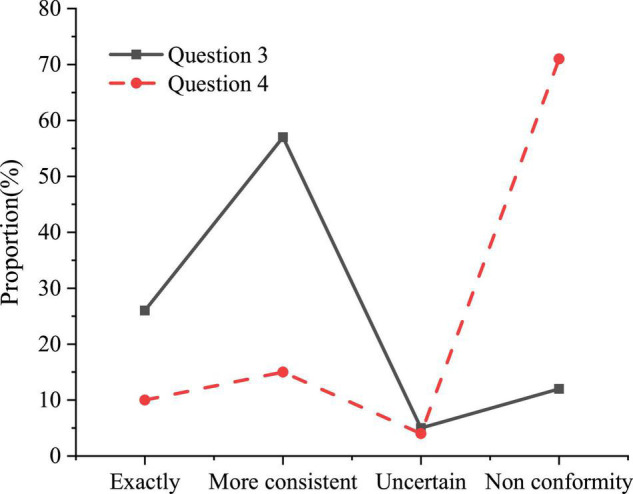
The results of the questionnaire on educational methods.

Nowadays, the teaching methods adopted by the college are relatively simple and lack novelty. Although a large number of scholars have put forward many feasible psychological quality education methods, they have not been specifically applied in educational activities. The results of the questionnaire of methods on psychological quality education in colleges are shown in Figure 3.

Question 3 refers to “the educational methods currently used in learning are mainly classroom lectures, psychological counseling and special lectures, etc.” and Question 4 refers to “the college has a psychological counseling telephone, website and mailbox, and actively conducts online counseling services and online counseling appointments.” Figure 3 indicates that 83% of the students think that the psychological quality education currently carried out by the college still adopts the more traditional teaching methods such as classroom lectures, psychological counseling and special lectures, and only 25% of the students believe that the college actively online consultation services and online consultation appointments are carried out. Some colleges do not pay enough attention to the course of psychological quality education, and the number of class hours set is relatively small. There is also a certain gap between teaching form, class schedule, content setting and other aspects and the requirements of formal curriculum construction.

#### The Teaching Staff Needs to Be Strengthened

**FIGURE 4:**
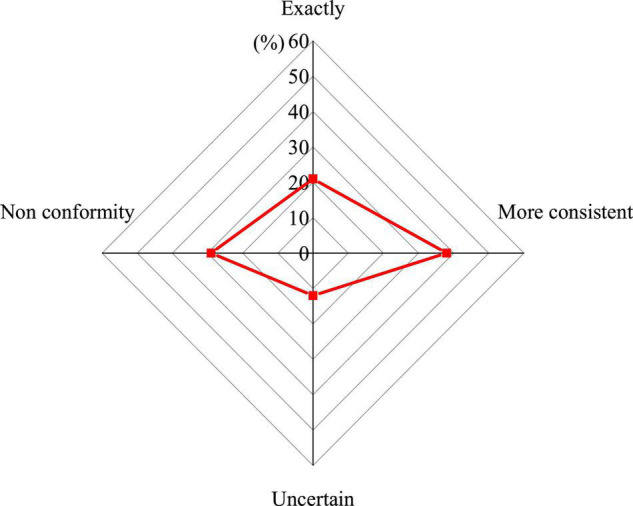
The professional level of the teaching staff of the colleges’ psychological quality education needs to be improved.

Psychological quality education requires educators to have high professional skills and a theoretical foundation. The results of the questionnaire on the professional level of the teaching staff of psychological quality education are shown in Figure 4.

Figure 4 shows that more than half of the students have a negative attitude toward the professional level of the teaching staff of the colleges’ psychological quality education teachers, indicating that at this stage, the teaching staff of China’s psychological quality education still lack professionalism. On the one hand, the number of psychological quality educators needs to be improved. Relevant documents clearly require the number of psychological quality educators in China, requiring all institutions of colleges to set up mental health counseling room, and to equip professional educators according to the teacher–student ratio of no less than 1:4,000. However, at present, it is relatively difficult for colleges to achieve this requirement. On the other hand, the professional level of psychological quality educators needs to be improved.

As Figure 4 suggests, 59% of students pointed out that the professional level of the teaching staff of the colleges’ psychological quality education needs to be improved. Now, it is mainly ideological and political workers, pedagogical or psychological teachers, administrative workers, and medical workers in school infirmary engaged in mental health education in colleges. Although these people have a strong sense of responsibility and rich work experience, they lack practical experience and professional knowledge and skills in educational psychology, and it is difficult to grasp the laws of students’ physical and mental development. When carrying out psychological counseling, it is mainly from the ideological and moral education level, and it is impossible to effectively deal with the psychological problems faced by college students from a professional perspective.

### Analysis of the Research Results on the Relationship Between Psychological Quality Education and Mental Health Level of College Students

#### The Basic Situation of Mental Health Level of College Students

**FIGURE 5:**
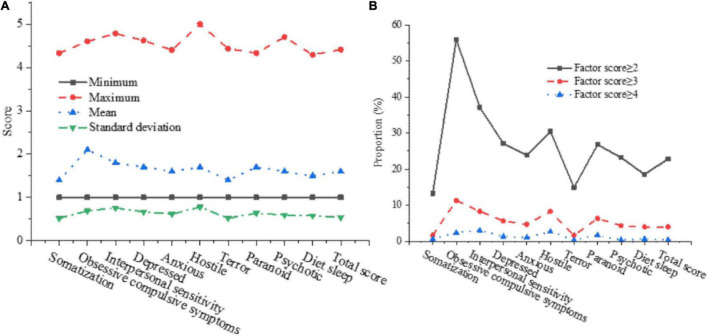
The statistical results of mental health level of college students. **(A)** Overall situation of mental health level. **(B)** Detection rate of mental health problems.

The score of each factor in the SCL-90 is counted, and the single factor score is screened to have a deeper understanding of the mental health level of college students. The statistical results are shown in Figure 5.

In Figure 5A, the average value of obsessive-compulsive symptoms from the SCL-90 has reached 2.15 scores, and the average value is the largest. After sorting the averages of each factor in the SCL-90, it is obtained: somatization < terror < diet and sleep < psychotic < anxious < paranoid < hostile < depressed < interpersonal sensitivity < obsessive-compulsive symptoms. The average score is 1.6, indicating that the psychological condition of most students is normal, but it still needs to pay attention to students with high mental health scores.

In Figure 5B, among the 383 college students surveyed this time, there are 87 students with a total factor score of ≥2 in the SCL-90, 15 students with a total factor score of ≥3, and only 1 student with a total factor score of ≥4. Through investigation and analysis, it is found that more than 20% of college students have certain psychological problems, and 0.33% of college students have serious mental health problems.

**FIGURE 6:**
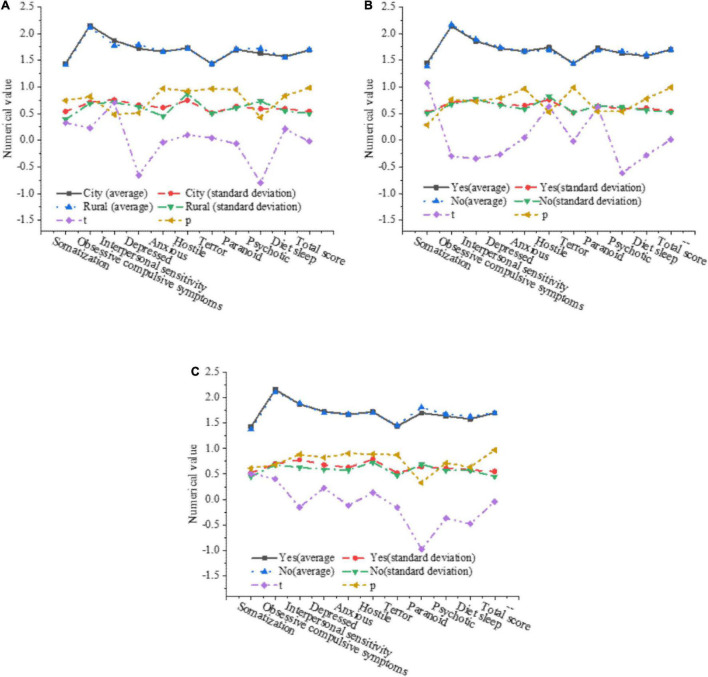
The influence of various factors on the mental health level of college students. **(A)** The influence of the birthplace on the mental health of college students. **(B)** The influence of whether it is the only child on the psychological health of college students. **(C)** The influence of single-parent families on the mental health of college students.

The influence of factors such as the birthplace, the only child and single-parent family on the mental health level of college students is further analyzed. The results are shown in Figure 6.

In Figure 6A, the birthplace has no significant impact on the mental health level of college students. In Figure 6B, the total score of both the only child and non-the only child is 1.7, and there is no obvious difference between factors, indicating that whether they are the only child will not have a significant impact on the mental health level of college students. In Figure 6C, the total average score of single-parent families and non-single-parent families is 1.7, and there is no significant difference between each factor, so it can be shown that whether it is a single-parent family will not have a significant impact on the mental health level of college students.

#### The Basic Situation of Psychological Quality Education of College Students

**FIGURE 7:**
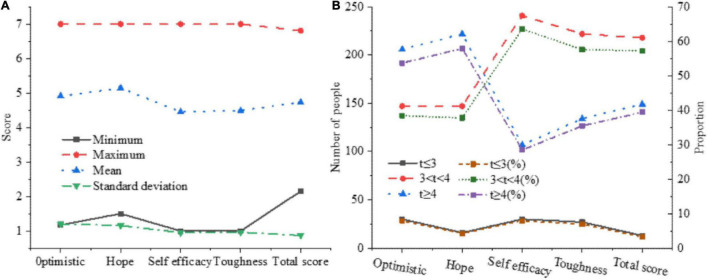
The statistical results of the psychological quality of college students. **(A)** Overall situation of psychological quality. **(B)** Detection rate of psychological quality.

The overall score of the questionnaire and each dimension are counted, and the number of people with scores of ≥4 and ≤3 in each dimension are screened, to have a deeper understanding of the overall psychological quality of college students. The statistical results are shown in Figure 7.

In Figure 7, more than half of college students’ psychological quality is average, nearly 40% of students’ psychological quality is positive, and 3.31% of college students’ psychological quality is negative, which needs to be paid attention to. The average of hope in the four dimensions has reached 5.14, and the average value is the highest. After sorting the average of each factor dimension in the questionnaire, you can get: resilience < self-efficacy < optimism < hope.

#### Correlative Analysis of Psychological Quality Education and Mental Health Level of College Students

**FIGURE 8:**
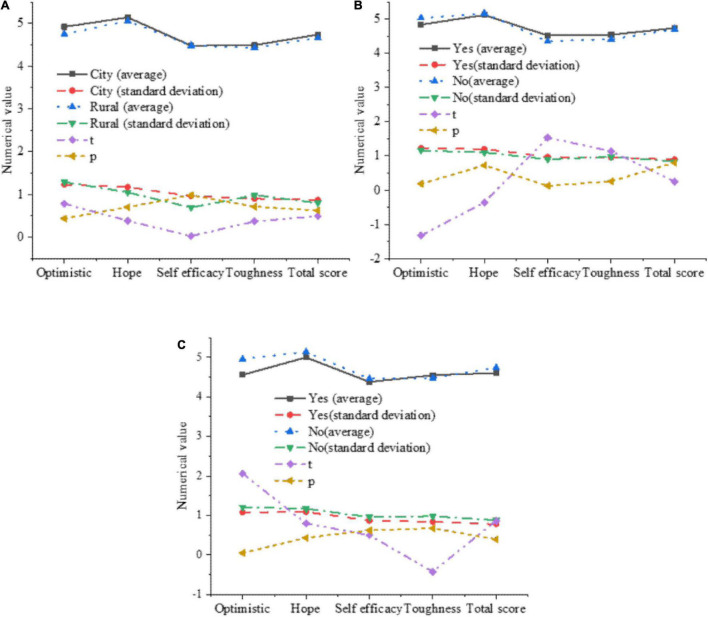
The influence of various factors on the psychological quality of college students. **(A)** The impact of the birthplace on the psychological quality of college students. **(B)** The impact of whether it is an only child on the psychological quality of college students. **(C)** The impact of single-parent families on the psychological quality of college students.

The influence of factors such as the birthplace, the only child and the single-parent family on the psychological quality of college students is further analyzed. The results are shown in Figure 8.

In Figures 8A,B, the birthplace and whether it is an only child will not have a significant impact on the psychological quality of college students. In Figure 8C, the optimism in the four dimensions will be significantly affected by single-parent families. The average value of college students growing up in single-parent families in the optimism is 4.56, while the average value of college students who grew up in non-single-parent families is 4.96, which is significantly higher than that of single–parent families.

#### Correlative Analysis and Regression Analysis of Mental Health Level in College Students’ Psychological Quality Qducation

According to the results of the questionnaire, the correlation between college students’ psychological quality education and mental health level is analyzed, and the results are shown in Table 1.

**TABLE 1:** Analysis of the correlation between college students’ psychological quality education and mental health level.

	Diet and sleep	Psychotic	Paranoid	Terror	Hostile	Anxious	Depressed	Interpersonal sensitivity	Obsessive-compulsive symptoms	Somatization
Optimism	–0.339**	–0.378**	–0.342**	–0.236**	–0.317**	–0.365**	–0.523**	–0.287**	–0.339**	–0.374**
Hope	–0.169**	–0.278**	–0.218**	–0.265**	–0.197**	–0.256**	–0.379**	–0.225**	–0.274**	–0.316**
Self-efficacy	–0.227**	–0.289**	–0.225**	–0.241**	–0.317**	–0.289**	–0.417**	–0.314**	–0.307**	–0.259**
Resilience	–0.289**	–0.417**	–0.307**	–0.269**	–0.431**	–0.328**	–0.508**	–0.466**	–0.371**	–0.284**

**p < 0.01.

In Table 1, there is a significant negative correlation between college students’ psychological quality education and mental health level, indicating that college students’ psychological quality education will have a relatively obvious positive effect on their mental health level, that is, psychological quality education will promote the development of college students’ mental health level. So, hypothesis H1 holds.

The stepwise regression is used to analyze the regression between college students’ psychological quality education and mental health level. The results are shown in Table 2.

**TABLE 2:** Analysis results of regression.

Stepwise regression	B	SE	R^2^	F	t
Resilience	–0.247	0.025	0.211	79.038	–8.870***
Resilience Optimism	–0.175 –0.121	0.031 0.029	0.264	51.825	–5.495*** –4.549***

***p < 0.001.

In Table 2, mental resilience first entered the regression equation, indicating that in the process of predicting the mental health level of college students, mental resilience plays an important role, and can predict 21.1% of the variables. Psychological resilience and optimism can jointly predict 26.4% of variables. Therefore, hypothesis H2 holds.

## Results and Discussion

The overall score of college students’ mental health demonstrates that college students’ psychological problems do not occur frequently. Obsessive-compulsive symptoms are more prominent in each factor, indicating that college students feel this symptom, and the severity is mild to moderate. The symptoms are thoughts and impulses that repeatedly intrude into the daily life of individuals, and they can experience these thoughts and impulses as originating from themselves, knowing that they are meaningless. Although they tried their best to resist, they are still unable to control it. The results manifest that the scores of girls on the dimension of fears are significantly higher than that of boys, indicating that girls are more likely to be psychologically troubled by terror. There is a stereotype of girls in many people’s ideas, thinking that girls are inferior to boys in university-level subjects, especially in subjects such as mathematics, physics and chemistry. Meanwhile, with the increasing pressure of further studies, girls are often in a state of tension, anxiety and unease, and their fears will become prominent. From the perspective of evolutionary psychology, girls are more delicate and sensitive than boys, and pay more attention to their inner experiences, so it is particularly significant to guide girls’ mental health.

The positive orientation of each dimension of psychological capital of young students, hope > optimism > resilience > self-efficacy, shows that more than half of the students have positive psychological capital that is relatively optimistic and hopeful. On the two dimensions of self-efficacy and resilience, scores between 3 and 4 are the most. In the traditional teaching method, due to the single assessment system and the low teacher-student ratio, many students have poor learning and character development, and lack of successful experience, which may affect the self-efficacy of college students. Influenced by factors such as interpersonal relationships, academic stress and other stressful events and the level of social support, the development of students’ psychological resilience will be affected.

## Conclusion

Psychological quality education is a significant part of ideological and political education. The party and the country attach great importance to the mental health and psychological quality training of college students. The successful holding of the “Positive Psychology and Construction of a Harmonious Society” International Forum in 2010 pushed mental health education to a higher historical starting point and height, and provided a good opportunity for the development of college students’ mental health education from the perspective of educational psychology.

Under the guidance of educational psychology, the current problems in the process of psychological quality education are investigated and analyzed in combination with new problems and situations. Firstly, through questionnaires, the data of college students’ psychological quality education and mental health level are collected. Through the processing of existing data, the general situation and relationship between college students’ psychological quality education and mental health level are analyzed and discussed.

The relationship between college students’ psychological quality education and mental health level is studied by means of a questionnaire. However, there are still some shortcomings in the research process. Psychological quality is the state of individual growth and development, and the state is unstable and may be affected by other factors and change. Psychological quality and mental health level are long-lasting and dynamic processes, so whether the effect of the intervention is sustainable and how long the impact on students can last needs to be tracked and investigated.

## Data Availability Statement

The raw data supporting the conclusions of this article will be made available by the authors, without undue reservation.

## Ethics Statement

The studies involving human participants were reviewed and approved by Hunan University Ethics Committee. The patients/participants provided their written informed consent to participate in this study. Written informed consent was obtained from the individual(s) for the publication of any potentially identifiable images or data included in this article.

## Author Contributions

The author confirms being the sole contributor of this work and has approved it for publication.

## Conflict of Interest

The author declares that the research was conducted in the absence of any commercial or financial relationships that could be construed as a potential conflict of interest.

## Publisher’s Note

All claims expressed in this article are solely those of the authors and do not necessarily represent those of their affiliated organizations, or those of the publisher, the editors and the reviewers. Any product that may be evaluated in this article, or claim that may be made by its manufacturer, is not guaranteed or endorsed by the publisher.

## Appendix

### Questionnaire Survey on Mental Health Education of College Students From the Perspective of Positive Psychology

Dear classmates:

Hi! This is a questionnaire about college students’ mental health education from the perspective of positive psychology. Please judge the degree of conformity of the following situations to you according to the actual situation, and tick “√” on the corresponding option. There is no right or wrong answer, and the survey results will be kept strictly confidential and used for academic research only. Please fill in carefully, thank you!

Your gender: A male B female

Your grade: A freshman B sophomore C junior D senior

Your major: A liberal arts B science C other

**Table:**

Questions	Choice			
	Exactly Compliant	Relatively Compliant	Not Compliant	Uncertain
1. I have some knowledge about positive psychology.				
2. I think that positive psychological qualities such as self-confidence and optimism are beneficial to the growth of college students.				
3. I think my mental state is healthy.				
4. I think it is necessary for colleges and universities to carry out mental health education.				
5. The school offers mental health education courses (elective or compulsory), and gives corresponding assessments and credits.				
6. The school can ensure that students in school generally receive mental health education.				
7. I think the philosophy of mental health education in schools is positive.				
8. The content of the school’s mental health education focuses on the prevention and resolution of students’ psychological problems.				
9. The school attaches great importance to mental health education, and can offer courses related to mental health education in combination with the actual situation.				
10. The school mainly adopts classroom lectures, special lectures, and psychological counseling to carry out mental health education.				
11. In addition to the theoretical courses of mental health education, schools often carry out various forms of mental health education and practical activities.				
12. The school’s mental health education and psychological counseling institutions have sufficient teachers.				
13. Do you think the professional level of the school’s mental health education faculty needs to be improved.				
14. The school has a psychological consultation mailbox, consultation telephone and website, and actively carries out online consultation appointments and online consultation services.				
15. I think that only students with psychological problems should have mental health education and psychological counseling.				
16. When I have psychological problems, I am willing to take the initiative to seek the help of teachers in the psychological counseling room.				
17. When I have psychological confusion, I am most willing to ask my close friends for help.				
18. I think the mental health education classes at school helped me grow.				
19. I am satisfied with the mental health education practice in the school.				
20. My parents took my mental health very seriously and were able to educate me in the right way.				
21. I believe that the social environment has an important influence on the development of my mental health.				
